# Supplementary material for: Droplet epitaxy symmetric InAs/InP quantum dots for quantum emission in the third telecom window: morphology, optical and electronic properties
Source: Nanophotonics. 2022 Jan 28;11(8):1515–26. doi: 10.1515/nanoph-2021-0482 (PMC11501369; doi:10.1515/nanoph-2021-0482)
Supplement: Supplementary file 1 — Supplementary Material Details [file j_nanoph-2021-0482_suppl.pdf]

Supplemental Material: Droplet epitaxy symmetric InAs/InP quantum dots for quantum emission in the third telecom window: morphology, optical and electronic properties

S-I. EDX DATA

In this section we show the raw EDX data which form the basis for the EDX profiles plotted in Fig. 2g for the QD and Fig. 2i for the 2D layer.

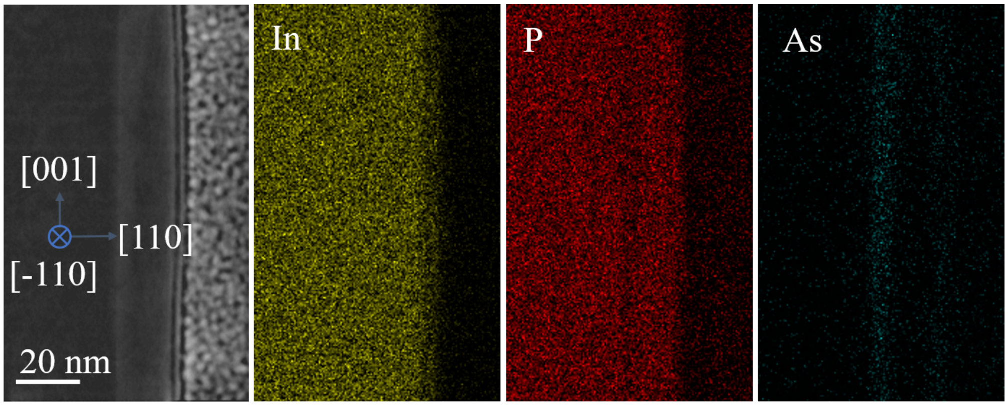

FIG. S1. EDX data for a QD viewed along the [-110] direction.

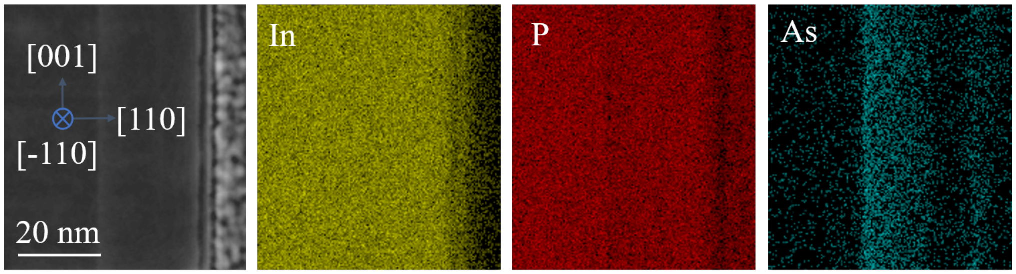

FIG. S2. EDX data for the 2D layer viewed along the [-110] direction.

S-II. RESULTS OF THE PIT ETCHING MODELS.

The results of QD-induced etching of InP are summarized in Fig. S3. Assumed geometry of the 2D layer is shown with the solid lines while the dashed lines show the results of the model, described in the text.

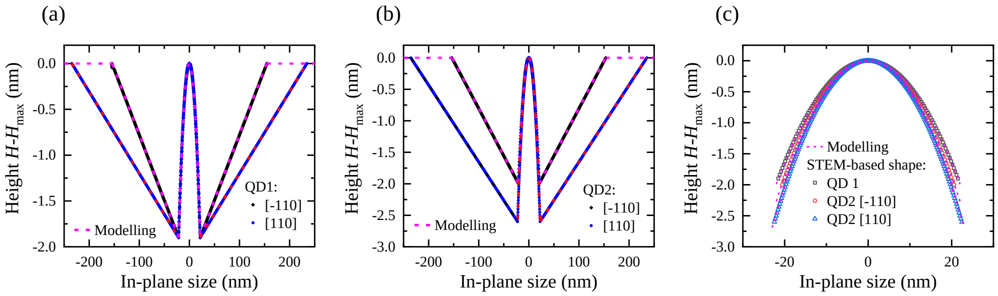

FIG. S3. Comparison of the assumed geometry of the pits and the 2D layer with the results of kinetic theory modelling for (a) the symmetric QD (QD1), (b) realistic QD with a slight asymmetry (QD2). (c) The close-up for the bottom of the QD.

S-III. CALCULATION OF EXCITONIC COMPLEXES IN QDS

Fig. S4 presents the results of binding energy calculations for a broad range of QD composition (As content, x = 72-88 %) and QD height (H = 3.6-6 nm).

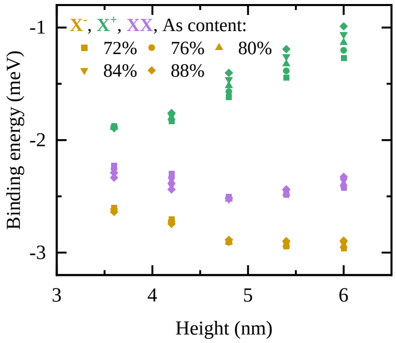

FIG. S4. Calculated binding energies for excitonic complexes confined within QDs as a function of QD height and As content.
